# ICU Admission Screening for Multidrug-Resistant Organism Colonization and Subsequent Infection Risk: A Pilot Study

**DOI:** 10.3390/diagnostics16142221

**Published:** 2026-07-16

**Authors:** Patricia Ionitoiu Chirodea, Stelian Adrian Ritiu, Marius Papurica, Dorel Sandesc, Daiana Toma, Adelina Baloi, Monica Licker, Corina Musuroi, Paula Irina Barata, Ovidiu Bedreag

**Affiliations:** 1 Faculty of Medicine, “Victor Babeș” University of Medicine and Pharmacy, 300041 Timișoara, Romania; patricia.chirodea@umft.ro (P.I.C.); stelian.ritiu@umft.ro (S.A.R.); daiana.toma@umft.ro (D.T.); adelina.baloi@umft.ro (A.B.); bedreag.ovidiu@umft.ro (O.B.); 2 Clinic of Anaesthesia and Intensive Care, Emergency County Hospital “Pius Brînzeu”, 300723 Timișoara, Romania; 3 Doctoral School, “Victor Babeș” University of Medicine and Pharmacy Timisoara, Eftimie Murgu Square 2, 300041 Timisoara, Romania; 4 Anaesthesia and Intensive Care Research Center (CCATITM), “Victor Babeș” University of Medicine and Pharmacy, 300041 Timișoara, Romania; 5 Microbiology Department, Multidisciplinary Research Center on Antimicrobial Resistance, ‘Victor Babes’ University of Medicine and Pharmacy, 300041 Timisoara, Romania; licker.monica@umft.ro (M.L.); corina.musuroi@umft.ro (C.M.); 6 Microbiology Laboratory Pius Brinzeu Emergency Clinical County Hospital (for Licker and Musuroi), 500723 Timisoara, Romania; 7 Department of Physiology, Faculty of Medicine, “Vasile Goldis” Western University of Arad, Blvd. Revolutiei, No. 96, 310025 Arad, Romania; barata.paula@uvvg.ro

**Keywords:** multidrug-resistant organisms, intensive care unit, hospital-acquired infection, admission screening, carbapenem-resistant Enterobacterales, antimicrobial resistance

## Abstract

**Background:** MDRO colonization at ICU admission may identify patients at increased risk for hospital-acquired infection (HAI), but its independent predictive value remains incompletely defined. **Methods:** We conducted a retrospective observational study of 560 consecutive ICU admissions (July–October 2024). Admission screening assessed colonization with extended-spectrum beta-lactamase (ESBL)-producing Enterobacterales, carbapenem-resistant Enterobacterales (CRE), and vancomycin-resistant enterococci (VRE). The primary outcome was microbiologically confirmed HAI; the secondary outcome was in-hospital mortality. Associations were evaluated using multivariable logistic regression, ROC/AUC analysis, mediation analysis, and a sensitivity analysis restricted to first admissions. **Results:** Of 560 admissions, 223 were screen-positive and 283 screen-negative. HAI occurred in 49 patients (8.8%). Positive screening was associated with increased HAI odds, narrowly missing significance in the primary adjusted analysis (OR 1.81, 95% CI 0.96–3.41, *p* = 0.065) but reaching significance in the first-admission sensitivity analysis (OR 2.09, *p* = 0.034). CRE showed the strongest adjusted association with HAI (OR 2.33, 95% CI 1.21–4.51, *p* = 0.012). Screening alone showed poor discrimination (AUC 0.574); combined with clinical variables, discrimination improved substantially (AUC 0.788). **Conclusions:** MDRO admission screening was associated with increased HAI odds, particularly for CRE, though screening alone has limited predictive performance. Larger prospective studies are needed to confirm these findings.

## 1. Introduction

Intensive care units (ICUs) represent the highest-risk environment for healthcare-associated infections (HAIs), defined as infections that develop after hospital admission and were neither present nor incubating at the time the patient was admitted. According to the WHO’s 2022 Global Report on Infection Prevention and Control [1], approximately 30% of patients hospitalized in adult ICUs acquire at least one HAI during their stay a rate nearly four times higher than that observed in general wards. These infections substantially increase morbidity, prolong hospitalization, and are independently associated with increased mortality, placing a considerable burden on both patients and healthcare systems [2].

A key driver of HAI risk in the ICU is the presence of multidrug-resistant organisms (MDROs)—bacteria that have developed resistance to multiple classes of antibiotics through three principal mechanisms: chromosomal mutations, acquisition of exogenous resistance genes (ARGs) via horizontal gene transfer, and clonal spread of resistant strains—making them particularly difficult to treat. Among the most clinically significant MDROs are extended-spectrum beta-lactamase-producing Enterobacterales (ESBL)—organisms that produce enzymes conferring resistance primarily against extended-spectrum cephalosporins (particularly 3rd generation) and monobactams (aztreonam), while carbapenem activity is generally preserved—carbapenem-resistant Enterobacterales (CRE), for which carbapenem resistance is the defining characteristic, and vancomycin-resistant enterococci (VRE). MDRO outbreaks have been steadily increasing in ICUs worldwide, yet healthcare institutions have not reached consensus on how and when to implement infection prevention and control strategies [1]. This lack of uniformity is consequential: a single CRE infection has been estimated to cost up to USD 63,948 per patient, and the downstream costs of uncontrolled outbreaks scale rapidly [3].

Active microbiological screening at ICU admission—the systematic collection of surveillance cultures from newly admitted patients to identify MDRO colonization before clinical infection develops—has been proposed as a cornerstone of infection control. The rationale is straightforward: a patient who carries a resistant organism on admission (colonization) is at higher risk of subsequently developing an active infection with that organism during their ICU stay. Identifying these patients early allows for targeted isolation measures and modified empiric antibiotic strategies. Despite this, most hospitals currently lack or choose not to implement universal MDRO screening programs upon ICU admission [4].

The evidence supporting the predictive value of admission screening, however, remains incompletely characterized. Several studies have examined MDRO colonization prevalence and associated clinical outcomes, but few have systematically evaluated whether a positive admission screen independently predicts HAI development after adjusting for confounding clinical factors—variables such as age, sex, and length of stay that may independently influence infection risk and that could explain an apparent association if not accounted for. Adjusted analysis (multivariable logistic regression—a statistical method that estimates the effect of one variable while holding others constant) is essential to isolate the true contribution of screening status. Studies investigating the association between MDRO colonization and clinical outcomes in ICU patients have reported low positive predictive values for standard surveillance cultures, raising questions about whether screening alone is sufficient as a risk stratification tool or whether it requires contextual clinical information to be actionable [5].

Furthermore, the relative impact of individual MDRO classes—ESBL, CRE, and VRE—has not been consistently compared within the same cohort. Each organism has a distinct transmission dynamic, resistance profile, and clinical trajectory. In terms of anatomical colonization sites, ESBL-producing Enterobacterales and CRE preferentially colonize the gastrointestinal tract and are predominantly detected from rectal or perineal swabs. VRE similarly colonizes the gastrointestinal tract and is detected from perineal specimens. Understanding these site-specific colonization patterns is essential for designing effective screening protocols. CRE, for instance, may be associated with rapid clinical deterioration and shorter ICU stays, whereas colonization with ESBL-producing Enterobacterales may confer a more gradual infection risk over longer hospitalizations. Co-colonization with multiple resistant organisms—a growing phenomenon in high-acuity settings—adds further complexity, as the additive or synergistic effects on HAI risk and mortality remain poorly defined [6].

A separate but equally important question is whether the pathway from MDRO colonization to death is direct or mediated through HAI. Mediation analysis—a statistical approach that decomposes an observed association into a portion that operates through an intermediate variable (in this case, HAI) and a portion that does not—can illuminate whether infection prevention alone is sufficient to reduce mortality, or whether colonization carries independent prognostic weight through mechanisms beyond infection [7].

Against this background, we conducted a retrospective observational study of 560 consecutive ICU admissions at a single center, in which systematic MDRO screening (ESBL, CRE, VRE) was performed at admission. The present study pursued five objectives. First, we examined whether a positive admission MDRO screen independently predicts the development of hospital-acquired infection after adjusting for age, sex, and length of stay. Second, we assessed whether admission screening status predicts in-hospital mortality and whether this association persists after multivariable adjustment. Third, we investigated whether the relationship between colonization status and mortality is mediated through HAI—that is, whether MDRO colonization increases mortality primarily by causing infection or through additional independent pathways. Fourth, we compared the independent risks for HAI and mortality conferred by each MDRO class (ESBL, CRE, and VRE) and evaluated whether co-colonization with multiple organisms confers additive risk. Fifth, we quantified the discriminatory value of admission screening—alone and in combination with clinical variables—for predicting HAI development, as measured by the area under the receiver operating characteristic curve (AUC), a metric ranging from 0.5 (no better than chance) to 1.0 (perfect discrimination).

## 2. Materials and Methods

### 2.1. Study Design and Data Source

This was a single-center retrospective observational study conducted in the ICU of Timișoara County Hospital. This study included exclusively general ICU admissions; burn unit patients were not included in the dataset. Data were extracted from the electronic medical record system for all consecutive admissions over the study period (July–October 2024). MDRO admission screening was performed as part of the standard clinical protocol for all patients admitted to the ICU at our institution. As this was a retrospective observational study using routinely collected clinical data, individual informed consent for screening was not required per applicable institutional guidelines. The study was conducted in accordance with the Declaration of Helsinki and was approved by the Institutional Review Board of “Pius Brânzeu” Emergency Clinical County Hospital Timișoara (approval number 569/08.10.2025).

### 2.2. Microbiological Screening Protocol

Admission MDRO screening cultures were collected from four anatomical sites: nasal swab, perineal swab, axillary swab, and inguinal swab. CRE, ESBL, and VRE screening was performed from perineal specimens. All cultures were processed using standard manual microbiological methods in the Microbiology Laboratory of “Pius Brânzeu” Emergency Clinical County Hospital, Timișoara. In the microbiology laboratory, as part of the routine workflow for microbiological diagnosis, chromogenic media (Thermo Fisher Scientific, Oxoid Deutschland GmbH, Wesel, Germany) were used to screen for colonization with ESBL-producing Enterobacterales, carbapenem-resistant Enterobacterales (CRE), and vancomycin-resistant enterococci (VRE). The screening method was designed solely to detect categories of MDROs, rather than to identify bacteria at the species level. The purpose of this approach was to assist clinicians in identifying potential treatment options for patients suspected of being colonized with ESBL-producing Enterobacterales, carbapenem-resistant Enterobacterales, or vancomycin-resistant enterococci.

For the detection of ESBL-producing Enterobacterales, Brilliance™ ESBL Agar (Thermo Fisher Scientific, Oxoid Deutschland GmbH, Wesel, Germany) was used. It is a qualitative selective chromogenic medium used to screen for the presence of extended-spectrum beta-lactamase (ESBL)-producing Enterobacterales and even *Acinetobacter* spp. in fecal and rectal screening samples. It contains cefpodoxime, in combination with additional antibacterial agents, to inhibit non-ESBL Enterobacterales and to suppress the growth of most bacteria that produce AmpC β-lactamase. After sample processing, plates have been incubated aerobically for 18–24 h at 35 ± 2 °C, according to the manufacturer’s recommendations. The presence of blue, pink, green, colourless, or brown colonies indicates the presence of presumptive ESBL-producing isolates, and colony color was used for presumptive species identification, in accordance with the manufacturer’s instructions. Blue or pink colonies indicate *E. coli*. Green colonies indicate *Klebsiella*, *Enterobacter*, *Serratia* and *Citrobacter* (KESC). Colourless colonies indicate *Salmonella*, *Acinetobacter* or other, while brown colonies with a halo indicate *Proteus*, *Morganella*, or *Providencia*.

Rectal and faecal screening samples were also screened for carbapenem-resistant Enterobacterales (CRE) using Brilliance™ CRE Agar, a qualitative selective chromogenic medium. It includes a modified carbapenem at a level suggested by the European Committee on Antimicrobial Susceptibility Testing (EUCAST) and Clinical & Laboratory Standards Institute (CLSI), guaranteeing accurate results with a variety of carbapenemases, including the New Delhi Metallo-β-lactamase-1 (NDM-1). After sample processing, plates have been incubated aerobically for 18–24 h at 35 ± 2 °C, according to the manufacturer’s recommendations. The presence of blue or pink colonies indicates the sample is CRE positive. Blue colonies are presumptive for *Klebsiella*, *Enterobacter*, *Serratia*, and *Citrobacter* (KESC). Pale pink colonies are presumptive for *E. coli*, while white or colourless colonies indicate the presence of non-CRE organisms. Resistant *Proteus*, *Morganella* and *Providencia* species produce tan colonies with a brown halo through the tryptophan deaminase (TDA) reaction. However, Brilliance™ CRE Agar has been reported to show different sensitivities and specificities for various carbapenemase types, with lower performance for some OXA-48-producing isolates compared with KPC- or NDM-producing isolates [8]. Therefore, our screening results should be interpreted as detection of presumptive CRE colonization, and some carbapenemase-producing organisms may have been underdetected.

Brilliance™ VRE Agar, a qualitative chromogenic medium, was used for the detection of vancomycin-resistant *enterococci* (VRE) in rectal or fecal specimens. Differentiation between vancomycin-resistant *E. faecium* and *E. faecalis* is achieved by the inclusion in culture medium composition of two chromogens that are targeted by specific enzymes: phosphatase and α-galactosidase. Additional antibiotics are also present in the composition, which, in combination with vancomycin, suppress the growth of competing flora, including *E. gallinarum* and *E. casseliflavus*, both of which are intrinsically resistant to vancomycin through the chromosomally encoded VanC resistance mechanism. Incubation conditions were: 18–24 h, 37° ± 2 °C aerobically, according to the manufacturer’s recommendations. The presence of pink-purple or blue colonies indicates the sample is VRE positive. Pink-purple colonies indicate *E. faecium*. Blue colonies indicate *E. faecalis.* White or colourless colonies indicate the presence of non-VRE organisms.

### 2.3. Study Population

A total of 560 admissions (484 unique patients) were included. Patients with multiple admissions during the study period were retained in the primary analysis; a sensitivity analysis restricted to the first admission per patient (*n* = 484) was performed to verify that repeat admissions did not bias the results (see Section 2.7).

Inclusion criteria: all adult patients (≥18 years) admitted to the ICU during the study period with a documented admission MDRO screening result or a recorded reason for absence of screening.

Exclusion criteria applied at the outcome level: infections documented on or before the admission date were not classified as hospital-acquired; infections with a first positive culture date after the discharge date were similarly excluded, as these could not be attributed to the current admission.

### 2.4. Variable Definitions

Exposure—MDRO screening status: Admission screening was classified as positive (colonization with ESBL, CRE, and/or VRE detected), negative (no resistant organism detected), or missing (no screening performed or result not recorded). The “missing” group was retained for descriptive reporting but excluded from all comparative analyses, as their clinical profile—median age 82 years, median length of stay 1 day, 0% HAI rate—rendered them non-comparable to the screened population, suggesting a distinct clinical trajectory (e.g., rapid transfer). Colonization was defined as the presence of an MDRO detected on admission screening culture in the absence of clinical signs or symptoms of active infection at the time of sampling [9].

Primary outcome—Hospital-Acquired Infection (HAI): Defined as a microbiologically confirmed infection with a first positive culture date strictly after the admission date and on or before the discharge date. This binary variable (yes/no) was the primary endpoint for screening efficacy analyses.

Secondary outcome—In-hospital mortality: Defined as death occurring during the index admission, recorded as a binary variable.

Covariates: Age (years, continuous), sex (male/female, binary), and length of stay (LOS, days, continuous) were included as covariates in all adjusted models, as these are established confounders of both HAI risk and mortality in ICU populations.

### 2.5. Statistical Analysis

All analyses were performed using Python 3.12.1 with the pandas 2.3.3, numpy 2.4.3, scikit-learn 1.8.0, scipy 1.17.1, statsmodels 0.14.6, lifelines 0.30.3, seaborn 0.13.2, and matplotlib 3.10.8 libraries. A two-sided significance threshold of α = 0.05 was applied throughout. Results are reported as median with interquartile range (IQR) for continuous variables and as count with percentage for categorical variables.

Normality testing: Before choosing between parametric and non-parametric tests, continuous variables were tested for normal distribution using the Shapiro–Wilk test. All continuous variables (age, LOS) significantly deviated from normality (*p* < 0.001), so non-parametric methods were used throughout.

Univariate comparisons: Associations between categorical variables (screening status, HAI, mortality) were assessed using Fisher’s exact test—a method appropriate for comparing proportions between two groups, particularly when cell counts are small. Continuous variables were compared between groups using the Mann–Whitney U test, a non-parametric equivalent of the independent samples *t*-test that does not assume a normal distribution.

Multivariable logistic regression: To determine whether screening status independently predicts HAI and mortality—after accounting for the confounding effects of age, sex, and LOS—a multivariable logistic regression was performed. Logistic regression estimates the odds of a binary outcome (e.g., developing HAI: yes vs. no) as a function of multiple predictors simultaneously. Results are expressed as adjusted odds ratios (ORs) with 95% confidence intervals (CIs). An OR greater than 1.0 indicates increased odds of the outcome in the exposed group; an OR of 1.0 indicates no difference. Multicollinearity—the undesirable condition in which two predictors are so strongly correlated with each other that the model cannot distinguish their individual effects—was assessed using the Variance Inflation Factor (VIF). A VIF close to 1.0 indicates no collinearity; values above 5 are considered problematic. All predictors in the final models had a VIF of ≈1.0.

Survival analysis: Time-to-event analyses were performed using the Kaplan–Meier method, which estimates the probability of surviving (or remaining infection-free) over time while accounting for patients who leave the study before the event occurs (censored observations). Differences between Kaplan–Meier curves were assessed using the log-rank test. Two outcomes were analyzed: time from admission to first HAI, and overall survival, both stratified by screening status.

Pathogen-specific analyses: The above univariate and multivariable analyses were repeated separately for each MDRO (ESBL, CRE, VRE), treating each as a binary exposure variable. Pairwise comparisons between pathogens were performed using Fisher’s exact test with Bonferroni correction for multiple comparisons—a conservative adjustment that reduces the risk of false-positive findings when multiple tests are run simultaneously by dividing the significance threshold by the number of comparisons performed.

Co-colonization analysis: Patients were classified into mutually exclusive groups based on their combination of colonizing organisms (e.g., ESBL only, ESBL + CRE, ESBL + CRE + VRE, etc.). HAI rates and mortality were compared descriptively across these groups to assess whether simultaneous colonization with multiple MDROs confers an additive risk.

Mediation analysis: To evaluate whether the association between screening status and mortality operates through HAI—that is, whether colonized patients die more often because they develop nosocomial infections—a formal mediation analysis was conducted using the Baron-Kenny framework. This approach decomposes the total effect of screening on mortality into two components: the indirect effect (the portion mediated through HAI development) and the direct effect (the remaining association not explained by HAI). The statistical significance of the indirect effect was assessed using bootstrapping with 5000 resampling iterations—a technique that repeatedly resamples the dataset to build an empirical distribution of the indirect effect and derive confidence intervals without assuming a normal distribution. A 95% bootstrap confidence interval not including zero was considered evidence of significant mediation.

Discriminatory performance—ROC/AUC analysis: The ability of screening status to distinguish patients who develop HAI from those who do not was quantified using the area under the receiver operating characteristic (ROC) curve (AUC). The ROC curve plots the true positive rate (sensitivity—the proportion of actual HAI cases correctly identified) against the false positive rate (1 − specificity—the proportion of non-HAI cases incorrectly flagged) across all possible decision thresholds. The AUC summarizes this performance as a single number ranging from 0.5 (no better than chance) to 1.0 (perfect discrimination). AUC values were interpreted as follows: <0.6 non-discriminating, 0.6–0.7 poor, 0.7–0.8 acceptable, 0.8–0.9 good, >0.9 excellent. Three models of increasing complexity were compared: screening status alone; screening status combined with demographic variables (age, sex, LOS); and pathogen-specific colonization combined with demographic variables.

Retrospective power analysis: Given the relatively modest sample size, a post hoc power analysis was performed to characterize the statistical power achieved for each primary comparison, that is, the probability that the study would detect a true effect of the observed magnitude, if one exists. Power was calculated using the two-proportions z-test framework (Cohen’s h effect size), with α = 0.05 and a two-sided alternative. The minimum sample size required to achieve 80% power for each comparison was also estimated.

### 2.6. Sensitivity Analysis

To address the potential bias introduced by including multiple admissions from the same patient—which violates the statistical assumption of independent observations—all primary regression models were re-run on a dataset restricted to each patient’s first ICU admission (*n* = 484). Consistency of odds ratios and significance levels between the full and restricted datasets was used to evaluate the robustness of the primary findings.

### 2.7. Software

Statistical analyses were performed in Python 3.12.1 using the following libraries: pandas 2.3.3 (data management), scipy 1.17.1 (non-parametric tests), statsmodels 0.14.6 (logistic regression, VIF), lifelines 0.30.3 (Kaplan–Meier, log-rank test), and scikit-learn 1.8.0 (ROC/AUC, propensity score estimation). Figures were generated using matplotlib 3.10.8 and seaborn 0.13.2.

### 2.8. Generative AI Usage Statement

During the preparation of this manuscript, AI tools were used to assist with language editing and code development. All outputs were critically reviewed by the authors, and all scientific content remains the responsibility of the authors.

## 3. Results

### 3.1. Patient Characteristics

A total of 560 ICU admissions were included in the analysis, corresponding to 484 unique patients; 76 admissions (13.6%) represented repeat hospitalizations of the same patient. The overall cohort had a median age of 66 years (IQR 55–74), and 309 patients were male (55.2%). Median length of stay (LOS) was 3 days (IQR 2–7). Over the study period, 49 patients (8.8%) developed a hospital-acquired infection, and 227 (40.5%) died during their admission. The estimated APACHE II score was 15.

Of the 560 admissions, 223 (39.8%) had a positive MDRO screen at admission, 283 (50.5%) were screen-negative, and 54 (9.6%) had no screening result recorded. The unscreened group was markedly distinct from the rest of the cohort—median age 82 years, median LOS 1 day (IQR 1–2), 0% HAI rate, and 1.9% in-hospital mortality—and was excluded from all comparative analyses (see Section 2). The demographic and clinical characteristics of the screened population are summarized in Table 1.

Patients with a positive admission screen had a significantly longer LOS compared to screen-negative patients (median 4 vs. 3 days, Mann–Whitney U = 35,216, *p* = 0.017), and numerically higher rates of both HAI (12.6% vs. 7.4%) and in-hospital mortality (50.2% vs. 40.3%).

### 3.2. Screening Status and Hospital-Acquired Infection

On unadjusted analysis, positive admission screening was associated with higher odds of developing HAI compared to negative screening (OR 1.79, *p* = 0.069), though this did not reach the pre-specified significance threshold of 0.05. The direction and magnitude of the association were, however, consistent throughout all analyses.

After adjusting for age, sex, and LOS using multivariable logistic regression, positive screening status showed a positive but non-significant association with HAI (adjusted OR 1.81, 95% CI 0.96–3.41, *p* = 0.065). The adjusted estimate was consistent in direction with the unadjusted finding, and the magnitude remained clinically relevant, but did not cross the pre-specified α = 0.05 threshold. Of the covariates, only LOS reached significance in the adjusted model (OR 1.05 per additional day, 95% CI 1.03–1.07, *p* < 0.001), indicating that longer hospitalization independently increases infection risk regardless of screening status. Age and sex were not significant predictors (*p* = 0.737 and *p* = 0.781, respectively). No multicollinearity was detected among predictors (all VIF ≈ 1.0). Full model results are presented in Table 2.

**Figure 1:**
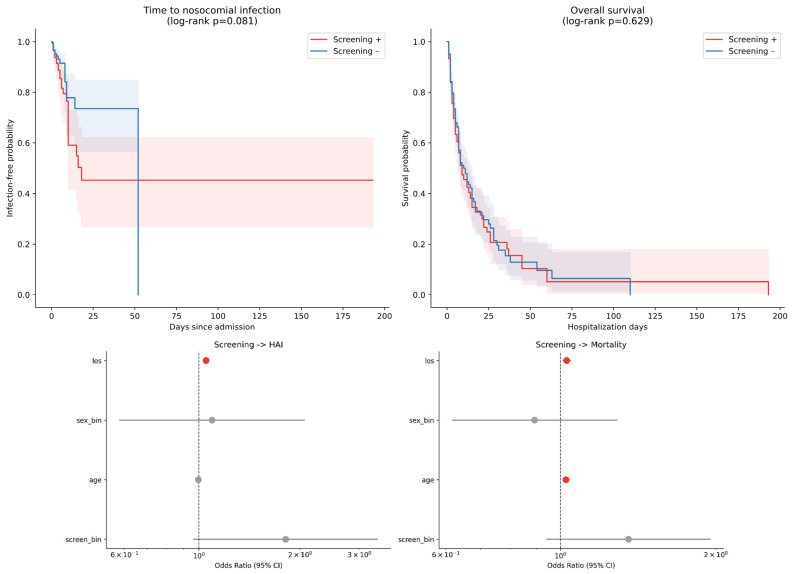
Time to event and adjusted regression analyses according to admission screening status.

Figure 1 shows a Kaplan–Meier analysis of time from admission to first HAI, showing a shorter median time to infection in screen-positive patients, with a borderline log-rank test result (*p* = 0.057), consistent with the univariate finding.

### 3.3. Screening Status and In-Hospital Mortality

On unadjusted analysis, screen-positive patients had significantly higher odds of in-hospital death compared to screen-negative patients (OR 1.51, *p* = 0.024). However, this association did not persist after multivariable adjustment for age, sex, and LOS (adjusted OR 1.36, 95% CI 0.94–1.96, *p* = 0.103). In the adjusted mortality model, older age (OR 1.03 per year, 95% CI 1.01–1.04, *p* < 0.001) and longer LOS (OR 1.03 per day, 95% CI 1.01–1.05, *p* = 0.003) were the only independent predictors of death. These results are summarized in Table 3.

Overall survival curves by screening status (Kaplan–Meier) did not differ significantly (log-rank *p* = 0.716), consistent with the non-significant adjusted regression result.

### 3.4. Pathogen-Specific Analyses

#### 3.4.1. Unadjusted Rates

Among the 223 screen-positive patients, ESBL was identified in 176 (31.4% of the total cohort), CRE in 157 (28.0%), and VRE in 31 (5.5%). HAI rates differed across pathogens: 13.6% in patients colonized with ESBL-producing Enterobacterales, 10.8% in those colonized with CRE, and 16.1% in those colonized with VRE. Mortality rates also varied substantially: 51.7% for ESBL, 34.0% for CRE, and 61.3% for VRE. CRE-colonized patients had a notably shorter median LOS (2 days) compared to ESBL and VRE patients (4 days each), suggesting more acute and rapidly evolving clinical trajectories in this subgroup.

#### 3.4.2. Adjusted Analyses

After adjusting for age, sex, and LOS, CRE colonization was a statistically significant independent predictor of HAI, while ESBL approached but did not reach significance. Patients colonized with ESBL-producing Enterobacterales had 1.85 times the odds of developing HAI compared to non-ESBL patients (95% CI 0.99–3.45, *p* = 0.055). CRE colonization showed the largest adjusted effect size for HAI (OR 2.33, 95% CI 1.21–4.51, *p* = 0.012), a notably larger effect than suggested by the unadjusted analysis—reflecting the confounding effect of CRE patients’ short LOS, which masked their true infection risk when LOS was not controlled for. VRE colonization showed a non-significant adjusted OR of 2.04 (95% CI 0.71–5.89, *p* = 0.188), likely attributable to insufficient statistical power given the small VRE subgroup (*n* = 31). No pathogen reached significance as an adjusted predictor of mortality.

### 3.5. Co-Colonization Analysis

Patients were classified into eight mutually exclusive groups based on their colonization pattern. HAI rates increased progressively with the number of co-colonizing organisms: 7.2% in screen-negative patients, 9.6% in ESBL-only, 19.4% in ESBL + CRE, and 25.0% in ESBL + CRE + VRE, suggesting an additive effect of co-colonization on infection risk. In contrast, combinations involving VRE exhibited paradoxically low HAI rates (0% for ESBL + VRE and CRE + VRE) alongside very high mortality (62.5% and 66.7%, respectively), consistent with a clinical pattern in which these patients die before a nosocomial infection has time to develop and be documented. CRE-only colonization was associated with the lowest HAI rate among colonized groups (2.5%), alongside low mortality (15.2%) and the shortest LOS (2 days). Full co-colonization data are presented in Table 4.

### 3.6. Mediation Analysis: Does HAI Explain the Effect of Screening on Mortality?

To determine whether the association between MDRO colonization and mortality operates through HAI development, a formal mediation analysis was conducted. For the primary model (overall screening status), colonization showed a non-significant association with HAI (path a: OR 1.82, *p* = 0.064), and HAI showed a positive but non-significant association with mortality after adjustment (path b: OR 1.45, *p* = 0.276). The direct effect of screening on mortality, independent of HAI, was also non-significant (adjusted OR 1.35, *p* = 0.113). The bootstrapped indirect effect—representing the portion of the screening-mortality relationship mediated through HAI—was 0.220 (95% bootstrap CI −0.269 to 0.813, *p* = 0.346), indicating that formal statistical significance for mediation was not achieved. The estimated proportion mediated was 70.6%, suggesting that, directionally, the majority of screening’s effect on mortality may operate through HAI, though this estimate carries wide uncertainty. Results were consistent across pathogen-specific mediation models. Notably, CRE showed a proportion mediated exceeding 100% (124.1%), a phenomenon known as suppression, in which the true effect of CRE on mortality is underestimated without accounting for HAI, consistent with CRE patients’ short LOS reducing their observed mortality rate.

### 3.7. Discriminatory Performance of Admission Screening

**Figure 2:**
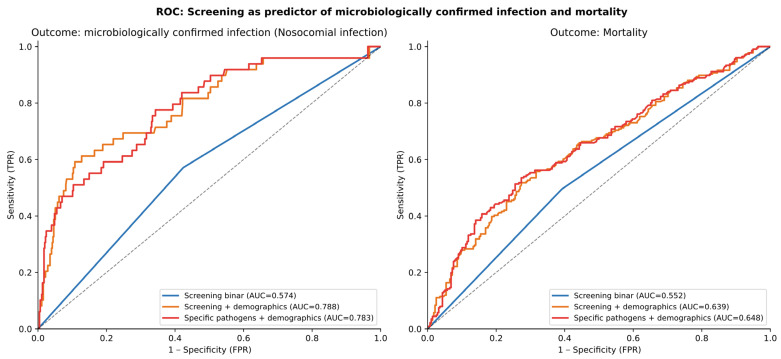
Discriminatory performance of MDRO screening models for HAI and mortality.

Models of increasing complexity were evaluated using ROC curve analysis to assess their retrospective discriminatory performance. Screening status alone showed poor discrimination for both HAI (AUC 0.574) and mortality (AUC 0.552), performing only marginally above chance. When combined with age, sex, and LOS, the model’s ability to discriminate HAI improved substantially (AUC 0.788), reaching acceptable discriminatory performance. Adding pathogen-specific identity (ESBL, CRE, VRE) rather than overall screening status did not meaningfully improve discrimination (AUC 0.783), indicating that what matters clinically is the presence of colonization, not which specific organism is detected. For mortality discrimination, no model achieved acceptable performance (maximum AUC 0.648), consistent with in-hospital mortality in ICU patients being driven by factors not captured in this dataset, such as illness severity scores and comorbidity burden. Results are shown in Figure 2. Because LOS is not available at admission, these models should be interpreted as retrospective discrimination models rather than real-time prediction tools.

### 3.8. Sensitivity Analysis: First Admission per Patient

To address the potential bias from including repeat admissions from the same patient, all primary regression models were re-run on a dataset restricted to each patient’s first ICU admission (*n* = 484). Outcome rates were virtually identical between the full and restricted datasets (HAI: 8.8% vs. 8.9%; mortality: 40.5% vs. 39.5%; median LOS: 3 days in both). Adjusted odds ratios were consistent in direction and magnitude across all models. Notably, the association between screening status and HAI reached statistical significance in the first-admission analysis (screening → HAI: OR 2.09, 95% CI 1.06–4.11, *p* = 0.034; screening →mortality: OR 1.50, 95% CI 1.01–2.24, *p* = 0.047) compared to the full dataset (screening →HAI: OR 1.81, *p* = 0.065; screening →mortality: OR 1.36, *p* = 0.103), suggesting that repeat admissions from surviving patients—a group with inherently lower mortality risk—slightly attenuate the associations in the full dataset (survivorship bias). These findings confirm the robustness of the primary results; the full dataset is reported throughout as the primary analysis, with first-admission results serving as a pre-specified sensitivity analysis.

### 3.9. Statistical Power

Post hoc power analysis revealed that the study was not adequately powered for any primary comparison. The primary comparisons of screening status with HAI and mortality had achieved power of 48.7% and 62.7%, respectively, reflecting the small effect sizes observed (Cohen’s h ≈ 0.17–0.21) relative to the available sample. The HAI →mortality comparison achieved 78.3% power, approaching but not reaching the 80% threshold. The ESBL→ HAI comparison achieved 75.2% power. These findings indicate that the non-significant results reported in this study—including the mediation analysis—should be interpreted as inconclusive rather than as evidence of the absence of effect, and that a sample size of approximately 527 patients per group would be required to detect the screening → HAI effect with 80% power.

## 4. Discussion

This retrospective observational study examined the relationship between MDRO admission screening status and two clinically critical outcomes—hospital-acquired infection and in-hospital mortality—in a cohort of 560 consecutive ICU admissions. Using a multi-level analytical framework that included adjusted regression, mediation analysis, ROC curve evaluation, and a pre-specified sensitivity analysis, we sought to characterize not only whether screening predicts outcomes, but through what mechanisms, to what degree, and with what discriminatory precision.

### 4.1. Screening as an Independent Predictor of HAI

The central finding of this study is that positive MDRO admission screening showed a consistent positive association with hospital-acquired infection across all analytical approaches, with an adjusted OR of 1.81 (95% CI 0.96–3.41, *p* = 0.065) that narrowly missed the pre-specified α = 0.05 significance threshold. While the association did not achieve formal statistical significance after multivariable adjustment, the direction and magnitude were stable throughout, and the first-admission sensitivity analysis did reach significance (OR 2.09, *p* = 0.034), suggesting that the full-dataset estimate is modestly attenuated by survivorship bias from repeat admissions. Colonization status therefore carries clinically plausible prognostic information, even if the current sample size was insufficient to establish it at conventional significance levels. Length of stay was the strongest individual predictor of HAI in the adjusted model (OR 1.05 per day, *p* < 0.001), which is biologically intuitive: longer exposure to the ICU environment increases cumulative infection risk regardless of colonization status. Importantly, screening and LOS act as independent rather than redundant predictors, as confirmed by the absence of multicollinearity between them (VIF ≈ 1.0 for all variables).

Both the unadjusted (OR 1.79, *p* = 0.069) and adjusted (OR 1.81, *p* = 0.065) associations between screening and HAI fell just short of statistical significance. This consistency across unadjusted and adjusted estimates is notable: unlike the CRE subgroup (Section 4.3), where adjustment substantially changed the effect magnitude, the overall screening estimate remained stable when LOS was controlled for, suggesting limited residual confounding at the aggregate level. The near-significant adjusted OR, combined with the significant first-admission sensitivity estimate (OR 2.09, *p* = 0.034), is consistent with a true association that is obscured by insufficient power rather than a true absence of effect. This pattern recurred in the ESBL subgroup analysis.

Several studies have demonstrated a high association between MDRO colonization and subsequent infections, particularly for organism-specific outcomes. Çalişkan Demirkiran et al. found in a study conducted on 118 patients that the infection rate with CRE pathogens in patients colonized at least once and in those without CRE colonization was 23.3% and 1.3%, respectively (*p* = 0.004). The incidences of sepsis (*p* = 0.003), septic shock (*p* = 0.01), intensive care unit transfer (*p* = 0.006), and 28-day mortality (*p* < 0.001) were significantly higher among patients colonized at admission [3]. In 2024, Guanhao et al. presented a retrospective study indicating that 25% of patients with carbapenem-resistant Enterobacterales (CRE) colonization subsequently developed an infection, negatively impacting their survival status over 60 days [10].

Sharma et al. conducted a study on 192 ICU patients who were screened for CRE. 37 patients were found to be colonized with CRE. *Klebsiella pneumoniae* (N = 25; 67.6%) was the most frequent CRE isolate, followed by *Escherichia coli* (N = 11; 29.7%) and one *Enterobacter* species (N = 1; 2.7%). 89.2% (33/37) of patients developed CRE infection. Pneumonia was the most common CRE infection identified in 12/33 (36.4%) patients during the hospital stay. The median duration of hospital stay was longer (17 days) for CRE colonized compared to CRE non-colonized patients (9 days) (*p* < 0.001). Death occurred in 27% (N = 10/37) of CRE-colonized patients during the hospital admission [11].

Our study found a more moderate association. The difference may be explained by the broader outcome definition for HAI used in our analysis. Another explanation for this difference can be represented by the differences in patient populations, ICU exposure and infection control practices.

### 4.2. Screening and Mortality: A Confounded Association

The unadjusted association between screening status and mortality was statistically significant (OR 1.51, *p* = 0.024), but this association attenuated to non-significance after multivariable adjustment (adjusted OR 1.36, *p* = 0.103). The independent predictors of mortality in the adjusted model were age (OR 1.03 per year, *p* < 0.001) and LOS (OR 1.03 per day, *p* = 0.003)—both established prognostic factors in critical care—while screening status did not contribute independently. This pattern strongly suggests that the crude association between screening and mortality is largely explained by confounding: screen-positive patients are older and stay longer, and it is these characteristics, rather than colonization per se, that drive their excess mortality.

This conclusion is further supported by the sensitivity analysis restricted to first admissions per patient (*n* = 484), in which the screening-mortality association reached significance (OR 1.50, *p* = 0.047). The difference between the two analyses is attributable to survivorship bias: patients who survive a first admission and are subsequently re-admitted are, by selection, a more resilient subgroup. Their inclusion in the full dataset dilutes the associations observed in the full analysis. The first-admission analysis, free of this bias, also showed a significant association between screening and HAI (OR 2.09, *p* = 0.034), suggesting that the true associations may be somewhat stronger than the primary analysis indicates.

Our findings are consistent with previous studies evaluating the prognostic role of MDRO colonization. In a cohort of patients with acute myeloid leukemia undergoing intensive chemotherapy, MDRO colonization was not associated with overall survival (HR 1.216, *p* = 0.301), mirroring our adjusted results. However, subgroup analyses in that study identified CRE colonization as a significant predictor of mortality (HR = 3.1), while other MDRO categories were not associated with adverse outcomes. This suggests that the impact of MDRO colonization on mortality may be pathogen-specific rather than a generalizable effect across all resistant organisms. In this context, our finding that screening positivity is not independently associated with mortality further supports the interpretation of MDRO colonization as a marker of patient vulnerability rather than a direct driver of mortality. Differences between studies may also reflect variations in patient populations, with hematological patients representing a particularly high-risk group in whom specific pathogens such as CRE may have more pronounced clinical impact. However, whether this magnitude of association is sufficient to justify universal screening remains debated and likely depends on local MDRO prevalence and available resources [12].

### 4.3. Pathogen-Specific Effects: ESBL, CRE and VRE Have Distinct Risk Profiles

The three pathogens examined in this study showed meaningfully different relationships with HAI and mortality, and treating them as interchangeable under a single “screen positive” label obscures important clinical heterogeneity.

Colonization with ESBL-producing Enterobacterales showed the most consistent positive association across all analyses—significant in the unadjusted analysis (OR 2.27, *p* = 0.009) and approaching significance in the adjusted model (OR 1.85, *p* = 0.055) for HAI. Although the adjusted estimate narrowly missed the α = 0.05 threshold, the direction and magnitude were stable, and the first-admission analysis confirmed significance (OR 2.02, *p* = 0.039). Of the three pathogens, ESBL had the largest subgroup (*n* = 176) and the highest achieved power (75.2%), making it the most robustly characterized finding of the study despite technical non-significance.

CRE demonstrated the largest adjusted effect size for HAI prediction (OR 2.33, *p* = 0.012), despite an unremarkable unadjusted rate (10.8% HAI, OR 1.41, *p* = 0.318). This discrepancy is explained by the confounding effect of LOS: CRE-colonized patients had a median LOS of only 2 days—half that of patients colonized with ESBL-producing Enterobacterales—which mechanically suppresses their observed HAI rate. Once LOS is held constant in the adjusted model, the underlying infection risk associated with CRE colonization becomes apparent. This is a methodologically important finding: crude HAI rates alone substantially underestimate CRE’s true predictive value in this cohort.

VRE presented a different pattern entirely. Despite the highest crude mortality (61.3%) and HAI rate (16.1%) among the three pathogens, no adjusted analysis reached significance, primarily due to the small subgroup size (*n* = 31, achieved power 66.3%). The co-colonization data offered a complementary observation: combinations involving VRE (ESBL + VRE: 0% HAI, 62.5% mortality; CRE + VRE: 0% HAI, 66.7% mortality) showed paradoxically absent HAI rates alongside extreme mortality, consistent with a clinical trajectory in which patients deteriorate and die before nosocomial infection has time to develop. Colonization with VRE may therefore signal extreme physiological vulnerability rather than infection risk per se.

In 2025, Woodhouse performed an umbrella review of systematic reviews and meta-analyses. An initial search for systematic reviews and meta-analyses yielded 847 results, with 17 articles ultimately included. After exclusion of 2 studies for overlapping results and very low quality, the pooled incidence of infection following colonization across the studies was 22% for ESBL-E and 22% for CRE [13]. Few reviews included high-quality findings on mortality or transmission following colonization. Additionally, only a limited number of reviews included findings related to MDR *Pseudomonas aeruginosa* or carbapenem-resistant *Acinetobacter baumannii*. The overall risk ratio for infection among colonized individuals was 16.41 compared with that without colonization (95% CI, 6.18–43.57). The risk ratio for infection following colonization with Enterobacterales was 15.83 compared with those without colonization (95% CI, 8.30–30.19). These findings have important implications for infection prevention and clinical management. In Europe, the European Centre for Disease Prevention and Control (ECDC) recommends differentiated infection prevention and control strategies for VRE, ESBL and CRE. In 2017, ECDC published dedicated guidance emphasizing active rectal screening on admission for at-risk patients, pre-emptive contact precautions and isolation. ESBL management relies primarily on a multifacet bundle of interventions, whereas VRE lacks a dedicated ECDC guidance document and is generally managed using standard precautions [9].

### 4.4. Co-Colonization: An Additive Gradient of Infection Risk

The co-colonization analysis revealed a clear stepwise increase in HAI rates with increasing numbers of co-colonizing organisms: 7.2% in screen-negative patients, 9.6% with ESBL alone, 19.4% with ESBL + CRE, and 25.0% with ESBL + CRE + VRE. This additive pattern suggests that each additional resistant organism independently contributes to infection risk, consistent with the hypothesis that co-colonization reflects cumulative immune compromise, prior antibiotic exposure, or repeated healthcare contact rather than a single exposure event.

The observation that CRE in isolation was associated with a notably low HAI rate (2.5%) and low mortality (15.2%)—lower even than screen-negative patients for mortality—is counterintuitive and warrants caution in interpretation. With *n* = 80 patients in this subgroup, random variation cannot be excluded. An alternative explanation is that CRE-only patients in this cohort represented a distinct clinical phenotype: possibly patients admitted for non-infectious reasons with incidental CRE colonization, or patients with very short stays who were discharged before infection could develop.

Our findings are in line with recent studies, which found that in ICU settings, colonization with multidrug-resistant bacteria is cumulative, increasing the duration of stay and preceding infection. In 2025, Garcia-Parejo H et al. found that of 7541 cases, 61.0% with initial colonisation had risk factors for MDROs versus 34.0% not colonized upon hospitalization (*p* < 0.001). Significant risk factors for initial colonization included hospitalization for ≥5 days within the last 3 months, prior MDROs colonization/infection and institutionalization. No significant risk factor differences were found for nosocomial colonization. An association between longer ICU stays and nosocomial colonization (*p* < 0.001) was noted [14].

Zhou et al. conducted a study from March 2016 to November 2018, 73 CRE bloodstream infections (BSIs) and 219 uninfected patients were included from 18 European hospitals [15]. For CRE versus CSE BSI, the previous CRE colonization/infection incidence rate ratio (IRR) 7.32; 95% CI 1.65–32.38) increased the risk. For CRE versus uninfected controls, independent risk factors included: older age (IRR 1.03; 95% CI 1.01–1.06), patient referral (long-term care facility: IRR 7.19; 95% CI 1.51–34.24; acute care hospital: IRR 5.26; 95% CI 1.61–17.11), previous colonization/infection with other MDR organisms (MDROs) (IRR 9.71; 95% CI 2.33–40.56), haemodialysis (IRR 8.59; 95% CI 1.82–40.53), invasive procedures (IRR 5.66; 95% CI 2.11–15.16), and β-lactam/β-lactamase inhibitor combinations (IRR 3.92; 95% CI 1.68–9.13) or third/fourth generation cephalosporin (IRR 2.75; 95% CI 1.06–7.11) exposure within 3 months before enrolment [14].

In 2025, Hernandez-Tejero M found that baseline rectal MDRO colonization was common in both the ward and ICU (23% vs. 17%; *p* = 0.38) and increased over time, reaching similar rates at 1 year (39% vs. 43%; *p* = 0.13). XDROs emerged during follow-up in both settings [16].

### 4.5. Mediation: HAI as a Partial but Unconfirmed Pathway to Death

The mediation analysis addressed whether HAI development explains the association between colonization and mortality, or whether colonization carries mortality risk through additional mechanisms. The results were directionally consistent—the estimated proportion of the screening-mortality association mediated through HAI was 70.6%—but did not achieve statistical significance (bootstrapped indirect effect 0.220, 95% CI −0.269 to 0.813, *p* = 0.346).

The reason for non-significance is mechanistically clear: path b (HAI → mortality, adjusted OR 1.45, *p* = 0.276) did not reach significance in any model, and formal mediation requires both paths to be reliably estimated. With only 49 HAI events in the cohort, the b-path is severely underpowered. The achieved power of 78.3% for the HAI →mortality comparison, while approaching the 80% threshold, was still insufficient. The CRE-specific mediation showed a proportion mediated exceeding 100% (124.1%), a suppression effect consistent with the LOS confounding described in Section 4.3: without HAI in the model, CRE’s true effect on mortality is systematically underestimated.

These findings do not refute mediation—they reflect an underpowered test of a plausible and directionally supported hypothesis. A study with at least 200–300 HAI events would be needed to test the mediation pathway with adequate power.

Similar observations can be found in the literature. In 2024, Botos et al. used the mNUTRIC score for a prospective, observational pilot study. A group of 46 patients with early septic shock, 42 nonseptic critically ill patients from the emergency department and 56 patients with late septic shock from the hospital were enrolled. On admission to the ICU, the most important potential predictors of 28-day mortality were assessed. A higher mNUTRIC score was the only common predictor for all three groups. Multi-drug resistant (MDR) bacterial etiology was a common predictor in both forms of septic shock. Older age, female gender, increased neutrophil-to-lymphocyte ratio (NLR), and increased need for vasoactive agents were common predictors in late septic shock and non-septic critically ill patients. Increased red blood cell distribution width coefficient of variation (RDW-CV) was a predictor in early septic shock and non-septic critically ill patients. Central venous-arterial carbon dioxide difference (Pcv-aCO_2_) was a predictor in patients with early septic shock. Inflammatory index and MDR carrier status were predictors in non-septic critically ill patients [17].

### 4.6. Discriminatory Performance: Screening Is Clinically Useful in Context

Admission screening status alone showed poor discriminatory performance for both HAI (AUC 0.574) and mortality (AUC 0.552). However, when combined with age, sex, and LOS, the model’s ability to predict HAI improved to AUC 0.788—an acceptable level of discrimination. Crucially, substituting overall screening status with pathogen-specific identity (ESBL, CRE, VRE as separate predictors) did not improve the AUC (0.783 vs. 0.788), indicating that what matters for prediction is the presence of colonization, not which organism is identified. This is practically relevant: it suggests that a binary positive/negative result at admission, interpreted alongside basic clinical variables, provides most of the available predictive signal.

For mortality prediction, no model exceeded an AUC of 0.648, confirming that in-hospital death in this cohort is driven by factors absent from the dataset—most likely illness severity, comorbidity burden, and the nature of the presenting diagnosis. This finding underscores the limitation of any model built on administrative data alone for mortality risk stratification.

The AUC of 0.789 for HAI prediction is slightly low, but remains within the range of clinically acceptable discrimination. Screening should not be implemented for predictive purposes but could be integrated into targeted infection prevention strategies, antimicrobial stewardship and outbreak control. It may provide meaningful clinical and economic benefits by reducing transmission and downstream complications.

### 4.7. Implications for Study Design: Sample Size and Power

A notable finding of this study is that it was not adequately powered for any of the five primary comparisons. This has two implications. First, the non-significant results reported here—particularly for VRE, for the mediation pathway, and for the screening-mortality and screening-HAI associations in the primary analysis—cannot be taken as evidence of the absence of an effect. The confidence intervals around these estimates are wide, and true clinically meaningful effects cannot be excluded. Second, the data from this study now provide precise effect size estimates (OR ≈ 1.81 for screening →HAI, Cohen’s h ≈ 0.17) that can be used to power future prospective studies appropriately. A target of approximately 527 patients per group would achieve 80% power for the primary screening →HAI comparison at the observed effect magnitude.

The OR of 1.81 is consistent with other results from studies conducted by Zhou et al. in 2024 [15] and from Hernandez-Tejero et al. in 2025 [16]. This shows that our results are clinically plausible but they may not be sufficient to promote universal screening from an economic perspective. Larger studies are warranted to prove the utility of admission screening.

### 4.8. Limitations

Several limitations of this study warrant consideration when interpreting the findings.

Retrospective design and unmeasured confounders. This was a retrospective observational study, which means that patients were not randomly assigned to screening groups and that the association between screening status and outcomes cannot be interpreted as causal. Most importantly, illness severity at admission—commonly quantified in ICU research using scores such as APACHE II or SOFA—was not available in the dataset. Since sicker patients are both more likely to be colonized with MDROs and more likely to die, residual confounding by severity cannot be excluded.

Sample size and statistical power. The study was not adequately powered for any of the five primary comparisons.

Screening upon admission was performed using validated chromogenic media designed to classify isolates into predefined categories of MDROs. The identification of microbial isolates at the species level, followed by antibiotic susceptibility testing, was an integral part of routine microbiological diagnosis performed on all other specimens collected from patients admitted to the ICU. However, species-level characterization and molecular confirmation are beyond the aim of the present investigation. We would also like to acknowledge that Brilliance CRE agar is a screening tool whose performance varies by carbapenemase type, with lower sensitivity reported for some OXA-48-like producers. Accordingly, our findings represent presumptive CRE colonization rather than confirmed carbapenemase-producing organisms.

Limited temporal coverage. The dataset encompasses only three consecutive months (July–September 2024) and does not allow assessment of seasonal variation.

HAI definition and ascertainment. Hospital-acquired infection was defined operationally using administrative date fields—the date of the first positive microbiological culture relative to admission and discharge dates. This approach does not incorporate the standard 48 h clinical threshold, nor does it account for the clinical context of each culture result (e.g., whether the culture represented true infection or contamination/colonization).

Small subgroup sizes for VRE and co-colonization patterns. The VRE subgroup comprised only 31 patients, resulting in wide confidence intervals and insufficient power for adjusted analyses.

Unscreened patients. Fifty-four admissions (9.5%) had no MDRO screening result recorded. The reasons for absent screening were not available in the dataset.

Single-center design. All data were collected from a single ICU at one institution, limiting the generalizability of the findings to other settings with different case-mix, MDRO prevalence, antibiotic stewardship practices, and infection control protocols. External validation in multi-center cohorts is necessary before these results can inform broad clinical policy.

Repeat admissions. The inclusion of 77 repeat admissions from the same patients in the primary analysis technically violates the statistical assumption of independent observations.

## 5. Conclusions

In our single-center, retrospective study, MDRO colonization detected on admission was associated with higher odds of HAI, although this did not reach statistical significance. Colonization with CRE had the strongest association with HAI, while screening alone had a poor discriminatory performance. This suggests that MDRO screening may be useful when combined with other clinical variables, but larger, multicenter, prospective studies are needed to assess whether MDRO screening has clinical and economic value.

## Figures and Tables

**Table 1:** Baseline characteristics stratified by admission screening status.

	All (*n* = 560)	Screen+ (*n* = 223)	Screen− (*n* = 283)	Unscreened (*n* = 54)
Age, years, median (IQR)	66 (55–74)	67 (56–75)	65 (52–73)	82 (82–82)
Male sex, *n* (%)	309 (55.2%)	114 (51.1%)	164 (58.0%)	31 (57.4%)
Length of stay, days, median (IQR)	3 (2–7)	4 (2–9)	3 (2–7)	1 (1–2)
Hospital-acquired infection, *n* (%)	49 (8.8%)	28 (12.6%)	21 (7.4%)	0 (0.0%)
In-hospital mortality, *n* (%)	227 (40.5%)	112 (50.2%)	114 (40.3%)	1 (1.9%)

Abbreviation: IQR, interquartile range.

**Table 2:** Multivariable logistic regression analysis of predictors of HAI.

Variable	Adjusted OR	95% CI	*p*
Screening positive	1.81	0.96–3.41	0.065
Age (per year)	1.00	0.98–1.01	0.737
Sex (male)	1.09	0.58–2.07	0.781
LOS (per day)	1.05	1.03–1.07	<0.001

Abbreviations: OR, odds ratio; CI, confidence interval; LOS, length of stay; HAI, hospital-acquired infection.

**Table 3:** Multivariable logistic regression analysis of predictors of in-hospital mortality.

Variable	Adjusted OR	95% CI	*p*
Screening positive	1.36	0.94–1.96	0.103
Age (per year)	1.03	1.01–1.04	<0.001
Sex (male)	0.87	0.62–1.29	0.434
LOS (per day)	1.03	1.01–1.05	0.004

Abbreviations: OR, odds ratio; CI, confidence interval; LOS, length of stay.

**Table 4:** Clinical outcomes according to colonization pattern.

Colonization Pattern	*N*	HAI, %	Mortality, %	Median LOS, Days
Screen negative	293	7.2%	40.6%	3
ESBL only	94	9.6%	50.0%	3
CRE only	80	2.5%	15.2%	2
ESBL + CRE	62	19.4%	50.0%	4
ESBL + CRE + VRE	12	25.0%	66.7%	3
ESBL + VRE	8	0.0%	62.5%	4
VRE only	8	25.0%	50.0%	5
CRE + VRE	3	0.0%	66.7%	2

Abbreviations: HAI, hospital-acquired infection; LOS, length of stay; ESBL, extended-spectrum beta-lactamase-producing Enterobacterales; CRE, carbapenem-resistant Enterobacterales; VRE, vancomycin-resistant *Enterococcus*. Small subgroup sizes should be interpreted cautiously.

## Data Availability

The data are encapsulated within this article. Further details can be obtained upon request from either the primary author or the corresponding author.
